# Screening of Rice (*Oryza sativa* L.) Genotypes for Salinity Tolerance and Dissecting Determinants of Tolerance Mechanism

**DOI:** 10.3390/plants13071036

**Published:** 2024-04-06

**Authors:** Tianxiao Chen, Yanan Niu, Changdeng Yang, Yan Liang, Jianlong Xu

**Affiliations:** 1 State Key Laboratory of Rice Biology, China National Center for Rice Improvement, China National Rice Research Institute, Hangzhou 310006, China; chentianxiao@caas.cn (T.C.); yangchangdeng@caas.cn (C.Y.); 2 State Key Laboratory of Crop Gene Resources and Breeding, Institute of Crop Sciences, Chinese Academy of Agricultural Sciences, Beijing 100081, China; yanan.niu@utas.edu.au; 3 Shenzhen Branch, Guangdong Laboratory for Lingnan Modern Agriculture, Agricultural Genomics Institute at Shenzhen, Chinese Academy of Agricultural Sciences, Shenzhen 518120, China

**Keywords:** *Oryza sativa*, salinity tolerance, physiological mechanism, principal component analysis, cluster analysis

## Abstract

Soil salinity imposes osmotic, ionic, and oxidative stresses on plants, resulting in growth inhibition, developmental changes, metabolic adaptations, and ion sequestration or exclusion. Identifying salinity-tolerant resources and understanding physiological and molecular mechanisms of salinity tolerance could lay a foundation for the improvement of salinity tolerance in rice. In this study, a series of salinity-tolerance-related morphological and physiological traits were investigated in 46 rice genotypes, including Sea Rice 86, to reveal the main strategies of rice in responding to salinity stress at the seedling stage. No genotypes showed the same tolerance level as the two landraces Pokkali and Nona Bokra, which remain the donors for improving the salinity tolerance of rice. However, due to undesirable agronomic traits of these donors, alternative cultivars such as JC118S and R1 are recommended as novel source of salinity tolerance. Correlation and principal component analyses revealed that the salinity tolerance of rice seedlings is not only controlled by growth vigor but also regulated by ion transport pathways such as long-distance Na^+^ transport, root Na^+^ sequestration, and root K^+^ retention. Therefore, such key traits should be targeted in future breeding programs as the strategy of obtaining better Na^+^ exclusion is still the bottleneck for improving salinity tolerance in rice.

## 1. Introduction

Crop yield and productivity are adversely affected by various biotic and abiotic stress in dynamic environments. Salinity stress is one of the most brutal abiotic stresses that reduce land and water productivity. Soil and water salinization can occur in both inland and coastal areas. Climate change and human practices also aggravate the salinization of soils, despite the fact that over 20% of irrigated land is salt-affected [1]. Soil salinity imposes osmotic, ionic, and oxidative stresses to plants, disrupting overall physiological, metabolic, and developmental activities and, thus, influencing the growth and survival of plants [2]. Rice (*Oryza sativa* L.) is one of the most preferred staple foods, feeding more than three billion people worldwide. However, cultivated rice is generally categorized as a typical glycophyte and is classified as a salinity-sensitive crop, especially during its seedling and reproductive stages [3,4]. Identifying promising rice germplasms with high salinity tolerance is fundamental for dissecting the physiological and molecular basis of salinity tolerance in rice and developing salinity tolerant rice cultivars. The selection of suitable and reliable criteria for assessing salinity tolerance is also a prerequisite for improving salinity tolerance in cultivated rice.

Salinity tolerance is expressed as the ability to grow and survive under salinity conditions [5]. Classical salinity tolerance evaluation approaches generally include morphological, physiological, and biochemical components. The Standard Evaluation System (SES) for Rice was proposed by The International Rice Research Institute (IRRI), scoring the salinity tolerance of rice from 1 to 9 based on the visual salt injury [6]. Lin et al. [7] believed that the survival day of seedlings (SDS) was a final criterion that measured the salinity tolerance of the rice seedlings. Under salinity stress, a dynamic competition takes place between Na^+^ and K^+^ for uptake as both are physico-chemically similar monovalent cations [8]. Also, strong membrane depolarization caused by Na^+^ uptake leads to K^+^ leakage from cells via depolarization-activated, outward-rectifying K^+^ channels [9]. The cytosolic K^+^/Na^+^ ratio has been repeatedly named as a key determination of plant salinity tolerance. Several studies on barley, wheat, and rice have suggested that the ability of root cells to retain K^+^ is crucial for salinity tolerance [10,11,12]. As a result of a better K^+^ retention in roots, salinity-tolerant genotypes have the ability to maintain higher K^+^/Na^+^ ratio in root cells, enabling better performance in saline conditions. Also, better vacuolar Na^+^ sequestration was found in salinity-tolerant wild rice species *Oryza rufipogon* and *Oryza coarctata* [13,14], demonstrating that vacuolar Na^+^ sequestration plays an important role in plant overall salinity tolerance [15]. Thus, the salinity tolerance of plants is an intricate phenomenon as it requires the combination of various independent and/or interdependent traits [16]. The quantification of salinity tolerance poses serious difficulties for practical breeding. So far, a range of morphological and physiological characters have been used to evaluate salinity stress tolerance in rice [17]. Morphological parameters based on overall performance, such as plant survival, salt injury score, and plant biomass, are preferred in most screening and breeding programs. Several basic physiological and biochemical parameters including chlorophyll content, Na^+^ and K^+^ concentration in shoots and roots, proline content, and sugars are also used to evaluate salinity tolerance in rice. Although salinity tolerance is controlled by polygene, most studies still treat salinity tolerance as a single trait and commonly use visual scoring or the Na^+^/K^+^ ratio for classification.

By screening thousands of rice genotypes for salinity tolerance, researchers have identified hundreds of genotypes that are highly salinity tolerant, including wild species *Oryza coarctata* and *Oryza glaberrima* accessions and cultivated landraces Pokkali and Nona Bokra [18]. Among the donor genotypes, the landraces Pokkali and Nona Bokra, and their derived breeding lines, remain the most popular donors of choice for breeding salinity-tolerant rice varieties. Sea Rice 86 (SR86) is reported to be domesticated from wild rice, which was first found in 1986 in sea-water-submerged, saline–alkaline soil near the coastal region in Zhanjiang, Southeast China. After more than 20 years of selection and domestication, it is generally believed that SR86 has retained the unique traits of abiotic stress tolerance and biotic stress resistance [19]. However, the high salinity tolerance of SR86 remains controversial, despite several physiological and genetic studies considered it to be salinity tolerant [20,21]. Here, we classified 46 rice genotypes including landraces and commercial varieties for salinity tolerance by comparing the change of several plant growth and ion concentration characters at seedling stage under salinity treatment and control conditions, in order to identify novel salinity-tolerant resources and to investigate the contribution of these traits to overall salinity tolerance in rice.

## 2. Results

### 2.1. Phenotypic Variation

The mean performances of the 46 rice genotypes for growth and ion concentration parameters under control and salinity stress are presented in Supplementary Table S1. All the characters showed considerable variations among the 46 rice genotypes (Table 1). Pokkali seedlings survived for the longest—25.3 days after exposure to 140 mM NaCl—while IR29 had an SDS of 10.1. On the 9th day post salinization, Pokkali had a mean salt injury score (SIS) of 2.3 and IR29 had a mean score of 9. Shoot growth was inhibited in all genotypes; the relative shoot fresh weight ranged from 23.45% to 76.72% and the relative shoot height ranged from 50.72% to 86.18%. But, interestingly, the root growth of several genotypes was slightly inhibited or even stimulated by salinity stress as the relative root length ranged from 73.31% to 170.63% and relative root dry weight ranged from 51.08% to 102.15%.

Varying concentrations of K^+^ and Na^+^ were observed among the 46 genotypes. The 9 days of salinization caused increased Na^+^ concentrations and reduced K^+^ concentrations in shoots and roots. In general, the shoot Na^+^ concentration (SNaC) was about three times the shoot K^+^ concentration (SKC), while the root Na^+^ concentration (RNaC) was more than six times the root K^+^ concentration (RKC). The shoot Na^+^ concentration was about three times the root Na^+^ concentration and the shoot K^+^ concentration was about five and half times the root K^+^ concentration. The relative shoot K^+^ concentration (RSKC) ranged from 24.99% to 68.44% and the relative root K^+^ concentration (RRKC) ranged from 6.24% to 37.47%, indicating that more K^+^ leakage happened in roots exposed to 140 mM NaCl. The landrace Nona Bokra had the lowest SNaC of 0.7 μM mg^−1^, highest SKNa of 0.98, highest RKC of 0.19 μM mg^−1^, and highest RRKC of 37.47%. Compared with Pokkali, a large amount of Na^+^ accumulation in shoots and massive K^+^ loss in roots were detected in IR29, leading to a worse K^+^/Na^+^ balance in both shoots and roots for IR29.

### 2.2. Trait Correlations

The correlation coefficients between SDS and plant growth parameters were presented in Table 2. Unsurprisingly, SDS showed a highly significant and negative correlation with SIS (*r* = −0.87, *p* < 0.001). Also, all the growth parameters under the saline condition were significantly and positively correlated with SDS. For the relative growth parameters, only the relative shoot fresh weight (RSFW) showed significant and positive correlation with SDS (*r* = 0.39, *p* < 0.01).

For ion-concentration-related traits, SNaC showed a significant and negative correlation with SDS (*r* = −0.66, *p* < 0.001), while no significant correlation was found between SKC and SDS (Table 3). SKNa showed a significant and positive correlation with SDS (*r* = 0.59, *p* < 0.001). Interestingly, SDS was significantly and positively correlated with RNaC (*r* = 0.46, *p* < 0.01), RKC (*r* = 0.69, *p* < 0.001), and RKNa (*r* = 0.55, *p* < 0.001). RRKC was also significantly and positively correlated with SDS (*r* = 0.65, *p* < 0.001). There was no significant relationship between SNaC and SKC. However, SNaC and RNaC was significantly and negatively correlated with RKC and RRKC.

### 2.3. Principal Component Analysis

**Figure 1:**
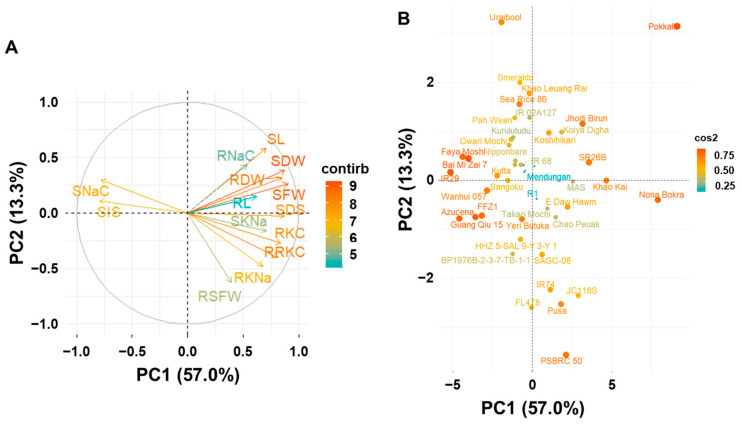
Principal component analysis plots: (**A**) traits factor plot; (**B**) genotype factor plot. All the parameters used for principal component analysis were investigated under salinity stress.

SDS and all the 13 parameters that showed significant correlations with SDS were used for principal component analysis and cluster analysis. The principal components (PC) analysis indicated that 70.3% of the total variance was represented by the two components (PC1 and PC2) with PC1 explaining 57.0% and PC2 explaining 13.3% (Figure 1A). All the traits were assigned to the positive side of PC1 except SIS and SNaC. Three main groups of traits are discernible: (1) total-biomass-related traits and RNaC in the first quadrant; (2) SNaC and SIS in the second quadrant; (3) ion-concentration-related traits in the fourth quadrant. These results suggest that the traits grouped together had significant correlations with each other. On the other hand, some traits like SNaC, SFW, RKC, and SKNa could be used to select high-salinity-tolerant genotypes.

The genotype factor plot showed that most salinity-tolerant genotypes like Pokkali, Nona Bokra, Khao Kai, and SR26B were assigned to the positive side of PC1, while most sensitive genotypes like IR29, Faya Moshi, and Azucena were assigned to the negative side of PC2 (Figure 1B).

### 2.4. Classification of 46 Rice Genotypes for Salinity Tolerance

**Figure 2:**
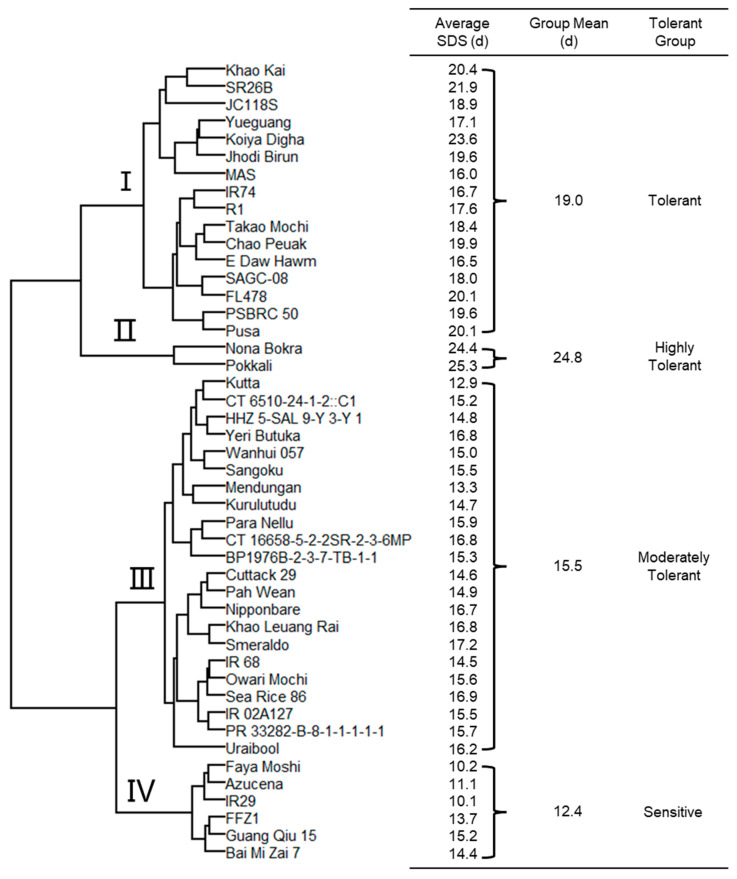
Clustering of 46 genotypes based on plant growth and ion concentration parameters in response to salinity stress.

The phenogram computed from the fourteen salt related traits produced four major clusters (Figure 2). Cluster Ⅱ was assigned as highly tolerant, grouping the two well-known landraces Pokkali and Nona Bokra, having an average SDS of 24.8. A total of 16 genotypes such as SR26B, FL478, and PSBRC 50 were classified as tolerant (cluster Ⅰ), having an average SDS of 19.0. Cluster Ⅲ consisting of 22 genotypes had a group SDS of 15.5 and was considered to be moderately tolerant. Six genotypes including IR29, Azucena, and Faya Moshi had an average SDS of 12.4 and, hence, were classified as sensitive (cluster Ⅳ).

## 3. Discussion

### 3.1. Identification of Salinity-Tolerant Resources

Considerable efforts have been made to evaluate diverse rice genotypes for salinity tolerance in the past few decades. Based on extensive screening, large variability in salinity tolerance has been identified in rice cultivars [4]. In the current study, twenty parameters including plant survival, growth vigor, and ion concentration were used to evaluate 46 rice cultivars for salinity tolerance at the seedling stage. Different parameters showed various rankings of genotypes in response to salinity stress, demonstrating wide phenotypic variation among rice cultivars. Also, high salinity tolerance could be achieved via the combination of multiple favorable traits under salinity stress. The landrace Sea Rice 86 (SR 86) found in coastal area has been a hot topic for years in China as several studies defined it as having high salinity tolerance [19,21]. However, SR 86 was classified as having moderate tolerance at the seedling stage (SDS = 16.9, SIS = 5.7) under 140 mM NaCl treatment. Interestingly, another two genotypes Guang Qiu 15 and Bai Mi Zai 7, which were phylogenetically closest to SR 86, were sensitive to salinity at the seedling stage. Hence, we still need to evaluate the potential of salinity tolerance in SR 86 thoughtfully and carefully, especially at the seedling stage.

Over the past few decades, landraces such as Pokkali and Nona Bokra are still the criterion by which the salinity tolerance of other rice genotypes is judged. Yeo and Flowers [22] argued that natural domestication and selection pressures for salinity tolerance in modern rice cultivars have been limited as salinity is marginal to the integrated ecological range of rice. It was speculated that identifying salt-tolerant traits in modern rice cultivars had reached a plateau as the real salinity tolerance traits in wild species may have been lost from long-term domestication [13,23]. In the current study, no genotypes showed the same tolerance level as Pokkali and Nona Bokra, which were selected as donors for improving salinity tolerance in rice. However, the drawbacks of using landraces as donor parents are negative “linkage drag” of undesirable traits such as photosensitivity, easy lodging, and low-yielding [24]. Based on cluster study, two breeding lines, JC118S and R1, were identified as salinity-tolerant genotypes. Our results show that JC118S has an SIS of 3.7 and an SDS of 18.9, while R1 has an SIS of 3.7 and an SDS of 17.6. JC118S is a commercial indica male sterility line with a high outcrossing rate and good combining ability for two-line hybrid rice. R1 is an elite restorer line for three-line hybrid rice. Therefore, JC118S and R1 can be used as novel sources of seedling salinity tolerance.

### 3.2. Salinity Stress Responsive Mechanisms in Rice

Salinity tolerance is expressed as the ability to grow and survive under salinity conditions. Our results show that growth vigor (SIS and shoot/root biomass) had strong correlations with survival under salinity stress, demonstrating that growth vigor is one of the major determinants of salinity tolerance in rice [5]. Also, shoot fresh weight under salt stress (SFW) showed a significantly negative correlation with SNaC, suggesting that the concentration of accumulated Na^+^ in the shoot was lower in fast growing cultivars than in slowly growing ones, mainly due to the dilution of sodium concentrations via growth. Hence, growth vigor is an avoidance mechanism that can alleviate the toxic effects of salinity stress [16]. In the present study, SDS showed significantly negative correlations with SNaC and RNaC, suggesting that control of xylem Na^+^ loading and its long-distance transport to shoot and root vacuolar Na^+^ sequestration could be one of the main mechanisms for salinity tolerance [25]. Despite no significant correlations being found between SDS, SKC, and RSKC, significantly positive correlations were detected between SDS, RKC, and RRKC. These results demonstrate that the ability to retain K^+^ in roots was one of the key traits conferring salinity tolerance [11,12,26]. The strategies for better Na^+^ extrusion from roots and better K^+^ maintenance in shoots have been employed in rice breeding programs for decades. Solis et al. argued that the strategies for obtaining better a Na^+^ exclusion or lower tissue Na^+^/K^+^ ratio in rice breeding has reached a plateau and cannot deliver any further improvement in salinity tolerance in this species [27]. Hence, our results call for better utilization of other traits such as root Na^+^ sequestration and root K^+^ retention in rice breeding programs.

### 3.3. Phenotyping Methodology

Over the past few decades, rice breeding has witnessed a revolutionary progress from conventional selection to molecular design breeding [28]. Meanwhile, the phenotyping methodology for salinity tolerance has made much slower progress. In the current study, we followed traditional phenotyping protocols [6] to evaluate 46 rice genotypes for salinity tolerance via a series of parameters based on the whole plant level. The strong correlations between SIS and growth-related traits we found were consistent with previous studies [29,30]. However, the correlations between survival under salinity (valued as SDS or SIS) and ion-concentration-related traits varied in different phenotyping systems. For instance, Lin et al. [7] and Rahman et al. [30] found that SDS and SIS showed no significant correlation with SKC, which is similar to our results, while a significant correlation between SIS and SKC was detected by De Leon et al. [29,31]. We speculate that the differences were mainly caused by a phenotyping error. This calls for precise phenotyping methodologies to evaluate such complex traits. Nondestructive high-throughput phenotyping platforms have been successfully applied in physiological and genetic studies of rice salinity tolerance and are believed to be promising strategies for large-scale phenotyping [32]. Also, tissue- and/or cell-based phenotyping strategy deserves greater attention [33,34].

## 4. Materials and Methods

### 4.1. Plant Materials

A total of 46 rice (*Oryza sativa* L.) genotypes were used to evaluate salinity tolerance and relevant characters at the seedling stage (see Table 4). The salinity-tolerant check FL478 and the salinity-sensitive check IR29 were also included. Seeds of Sea Rice 86, JC118S, and R1 were kindly provided by Risheng Chen. All other accessions were collected from the “3000 rice genomes project (3K RGP)” [35], and the seeds were germinated in Sanya, China.

### 4.2. Evaluation of Morphological and Physiological Traits for Salinity Tolerance

Evaluation protocols were conducted under hydroponic conditions, following the methods described previously [36]. The 96-well (12 × 8) PCR plates with perforate wells at the bottom were used for sowing, and 16 uniformly germinated seeds per genotype were sown in 2 rows (2 × 8) with an empty row between 2 genotypes. The materials were arranged in a complete randomized block design in three replications. These PCR plates were transferred into Yoshida’s nutrient solution after floating on water for 5 days. Fourteen-day-old seedlings were exposed to Yoshida’s solution containing 70 mM NaCl for 3 days to reduce immediate osmotic shock. Then, the saline solution was raised to 140 mM NaCl. The culture solutions were changed every third day, and the pH was maintained at 5.1 to 5.5. The phytotron was maintained on a regular basis (14 h of light, 28 °C, 70% relative humidity and 10 h of dark, 25 °C, 60% relative humidity). The survival days of seedlings (SDS) were recorded on a daily basis since the first plant died for each genotype.

To investigate morphological and physiological characters, a second experiment was performed. The procedure and management of the experiment was the same as the above-mentioned experiment. The second experiment was conducted in three replications with two treatments (control and salinity stress). The susceptible check IR29 showed characteristic salt toxicity symptoms 9 days after 140 mM NaCl treatment. Then, each genotype was given a visual salt injury score (SIS) of 1–9 following the IRRI Standard Evaluation System for Rice [6]. After removing the corner plants, only 8 plants of uniform growth per genotype of control and treatment were harvested for data collection. The seedling height (SH), root length (RL), shoot fresh weight (SFW), shoot dry weight (SDW), and root dry weight (RDW) of each genotype were subsequently measured. The dried samples of each genotype were extracted in 25 mL acetic acid (100 mmol L^−1^) at 90 °C for 2 h [37]. Shoot and root Na^+^ and K^+^ concentrations (SNaC, SKC, RNaC, and RKC) of each sample were determined via atomic absorption spectrometry (AAS, Series 2, Thermo Electron Corporation, Waltham, MA, USA) following the manual book. The K^+^/Na^+^ ratios in shoots (SKNa) and roots (RKNa) were calculated subsequently. Relative trait values were calculated according to the following formula: relative trait value (%) = (trait value under salt stress)/(trait value under control) × 100. The mean values of the traits for eight plants were regarded as one replicate.

### 4.3. Data Analysis

The mean value of each trait was used for statistical analysis. All statistical analyses of phenotypic data were performed using the SPSS software package (Version 28.0, IBM, Armonk, NY, USA). Principal component analysis and cluster analysis were conducted using R package “FactoMineR” (version 2.10) [38].

## 5. Conclusions

In this study, a series of salinity-tolerance-related morphological and physiological traits were investigated in forty-six rice germplasms at the seedling stage to reveal the main strategies of rice in responding to salinity stress. Abundant phenotypic variations among the forty-six rice genotypes were observed for salinity tolerance and related parameters. Although no genotypes showed the same tolerant level as Pokkali and Nona Bokra, two commercial varieties, JC118S and R1, were identified as being salinity tolerant at the seedling stage. We suggest that these two varieties may serve as novel donor parents for improving salinity tolerance in rice breeding programs. The results of correlation and principal component analyses revealed that the salinity tolerance of rice seedlings is not only controlled by growth vigor but also regulated by vacuolar Na^+^ sequestration and K^+^ retention in root cells. Our results imply that such traits can be considered as important determinants in rice salinity-tolerance screening and breeding programs. In conclusion, our findings highlight the importance of identifying novel salinity-tolerant rice resources and understanding dominant tolerance mechanisms.

## Figures and Tables

**Table 1:** Performances of plant-growth- and ion-concentration-related traits under control and salinity stress at seedling stages of the 46 rice genotypes.

Trial	Trait	Range	Mean ± SD
Control	SFW (mg)	96.10–777.40	256.17 ± 129.96
	SDW (mg)	15.83–107.40	38.43 ± 17.50
	RDW (mg)	4.23–16.93	7.65 ± 2.48
	SL (cm)	22.93–59.94	38.29 ± 9.47
	RL (cm)	5.29–15.57	8.87 ± 2.42
	SKC (μmol mg^−1^)	0.84–1.29	1.06 ± 0.10
	RKC (μmol mg^−1^)	0.35–0.72	0.51 ± 0.09
Salinity stress	SDS (d)	10.08–25.30	16.73 ± 3.25
	SIS	2.33–9.00	5.25 ± 1.92
	SFW (mg)	34.47–381.73	112.60 ± 62.32
	SDW (mg)	10.85–74.60	27.08 ± 10.95
	RDW (mg)	3.35–11.47	5.61 ± 1.57
	SL (cm)	12.84–43.71	24.93 ± 5.89
	RL (cm)	4.54–16.18	8.49 ± 2.17
	SKC (μmol mg^−1^)	0.28–0.71	0.56 ± 0.08
	SNaC (μmol mg^−1^)	0.70–2.44	1.74 ± 0.41
	SKNa	0.20–0.98	0.35 ± 0.14
	RKC (μmol mg^−1^)	0.04–0.19	0.10 ± 0.03
	RNaC (μmol mg^−1^)	0.48–0.87	0.64 ± 0.09
	RKNa	0.07–0.29	0.16 ± 0.05
Relative value	RSFW (%)	23.45–76.72	44.98 ± 12.71
	RSDW (%)	45.44–106.47	72.74 ± 14.88
	RRDW (%)	51.08–102.15	74.49 ± 13.81
	RSL (%)	50.72–86.18	65.70 ± 6.88
	RRL (%)	73.31–170.63	97.76 ± 20.38
	RSKC (%)	24.99–68.44	53.54 ± 9.36
	RRKC (%)	6.24–37.47	20.58 ± 6.49

SDS: survival days of seedling; SIS: salt injury score; SFW: shoot fresh weight; SDW: shoot dry weight; RDW: root dry weight; SL: seedling length; RL: root length; RSFW: relative shoot fresh weight; RSDW: relative shoot dry weight; RRDW: relative root dry weight; RSL: relative seedling length; RRL: relative root length; SNaC: shoot sodium concentration; SKC: shoot potassium concentration; SKNa: ratio of potassium and sodium concentration in shoot; RNaC: root sodium concentration; RKC: root potassium concentration; RKNa: ratio of potassium and sodium concentration in root; RSKC: relative potassium concentration in shoot; RRKC: relative potassium concentration in root.

**Table 2:** Pearson correlation coefficients between SDS and growth parameters.

	SDS	SIS	SFW	SDW	RDW	SL	RL	RSFW	RSDW	RRDW	RSL
SIS	−0.87 ***	1									
SFW	0.80 ***	−0.62 ***	1								
SDW	0.76 ***	−0.59 ***	0.98 ***	1							
RDW	0.65 ***	−0.54 ***	0.90 ***	0.91 ***	1						
SL	0.60 ***	−0.44 **	0.81 ***	0.86 ***	0.77 ***	1					
RL	0.40 **	−0.46 **	0.47 ***	0.48 ***	0.54 ***	0.50 **	1				
RSFW	0.39 **	−0.44 **	0.28	0.18	0.17	−0.1	0.1	1			
RSDW	0.17	−0.23	0.05	−0.02	0	−0.29 *	−0.05	0.92 ***	1		
RRDW	0.12	−0.15	0.10	0.03	0.12	−0.23	0	0.83 ***	0.85 ***	1	
RSL	0.21	−0.24	0.19	0.19	0.12	0.05	−0.12	0.59 ***	0.66 ***	0.51 ***	1
RRL	0.06	−0.07	0.04	−0.01	0.03	−0.14	0.29	0.6 ***	0.63 ***	0.70 ***	0.39 **

All the growth parameters were investigated under salinity stress; ‘*’, ‘**’, and ‘***’ refer to significant correlations (*p* < 0.05, *p* < 0.01, and *p* < 0.001).

**Table 3:** Pearson correlation coefficients between SDS and ion concentration parameters.

	SDS	SIS	SNaC	SKC	SKNa	RNaC	RKC	RKNa	RSKC
SIS	−0.87 ***	1							
SNaC	−0.66 ***	0.61 ***	1						
SKC	−0.03	−0.11	−0.09	1					
SKNa	0.59 ***	−0.43 **	−0.81 ***	0.53 ***	1				
RNaC	0.46 **	−0.45 **	−0.31 *	0	0.3 *	1			
RKC	0.69 ***	−0.66 ***	−0.63 ***	−0.14	0.51 ***	0.40 **	1		
RKNa	0.55 ***	−0.54 ***	−0.54 ***	−0.16	0.39 **	0.01	0.91 ***	1	
RSKC	−0.26	0.34 *	0.04	0.82 ***	0.34 *	−0.12	−0.27	−0.27	1
RRKC	0.65 ***	−0.58 ***	−0.68 ***	0	0.62 ***	0.30 *	0.85 ***	0.78 ***	−0.07

All the ion concentration parameters were investigated under salinity stress; ‘*’, ‘**’, and ‘***’ refer to significant correlations (*p* < 0.05, *p* < 0.01, and *p* < 0.001).

**Table 4:** The information of 46 germplasms used in the study.

Accession	Origin	Accession	Origin
Jhodi Birun	Bangladesh	Yueguang	Japan
Koiya Digha	Bangladesh	Takao Mochi	Japan
FFZ1	China	Sangoku	Japan
SAGC-08	China	Owari Mochi	Japan
R1	China	Chao Peuak	Laos
JC118S	China	Yeri Butuka	Nigeria
Sea Rice 86	China	Pusa	Pakistan
Wanhui 057	China	PR 33282-B-8-1-1-1-1-1	Philippines
Guang Qiu 15	China	IR 02A127	Philippines
Bai Mi Zai 7	China	IR 68	Philippines
CT 16658-5-2-2SR-2-3-6MP	Colombia	HHZ 5-SAL 9-Y 3-Y 1	Philippines
CT 6510-24-1-2::C1	Colombia	IR 29	Philippines
Nona Bokra	India	FL478	Philippines
Cuttack 29	India	Azucena	Philippines
SR26B	India	IR 74	Philippines
Para Nellu	India	PSBRC 50	Philippines
Uraibool	India	POKKALI	Sri Lanka
Kutta	India	Kurulutudu	Sri Lanka
BP1976B-2-3-7-TB-1-1	Indonesia	Faya Moshi	Tanzania
MAS	Indonesia	Khao Kai	Thailand
Mendungan	Indonesia	Khao Leuang Rai	Thailand
Smeraldo	Italy	Pah Wean	Thailand
Nipponbare	Japan	E Daw Hawm	Thailand

## Data Availability

The datasets supporting the conclusions of this article are included within the article and Supplementary Materials.

## Supplementary Materials

The following supporting information can be downloaded at: https://www.mdpi.com/article/10.3390/plants13071036/s1, Table S1: Mean performance of 46 rice genotypes for salinity characterization.
